# Yoga and Aerobic Dance for Pain Management in Juvenile Idiopathic Arthritis: Protocol for a Pilot Randomized Controlled Trial

**DOI:** 10.2196/12823

**Published:** 2020-07-06

**Authors:** Karine Toupin April, Jennifer Stinson, Sabrina Cavallo, Laurie Proulx, George A Wells, Ciarán M Duffy, Tania ElHindi, Patricia E Longmuir, Lucie Brosseau

**Affiliations:** 1 Children’s Hospital of Eastern Ontario Research Institute Ottawa, ON Canada; 2 Department of Pediatrics Faculty of Medicine University of Ottawa Ottawa, ON Canada; 3 Faculty of Health Sciences School of Rehabilitation Sciences University of Ottawa Ottawa, ON Canada; 4 Child Health Evaluative Sciences The Peter Gilgan Centre for Research and Learning The Hospital for Sick Children Toronto, ON Canada; 5 Lawrence S Bloomberg Faculty of Nursing University of Toronto Toronto, ON Canada; 6 École de Réadaptation Université de Montréal Montréal, QC Canada; 7 Canadian Arthritis Patient Alliance Ottawa, ON Canada; 8 Cardiovascular Research Methods Centre University of Ottawa Heart Institute Ottawa, ON Canada; 9 School of Epidemiology and Public Health University of Ottawa Ottawa, ON Canada; 10 Division of Rheumatology Children's Hospital of Eastern Ontario Ottawa, ON Canada; 11 Statistics Canada Ottawa, ON Canada; 12 School of Human Kinetics Faculty of Health Sciences University of Ottawa Ottawa, ON Canada

**Keywords:** juvenile idiopathic arthritis, yoga, aerobic exercise, dance, pain management, pilot

## Abstract

**Background:**

Juvenile idiopathic arthritis (JIA) is one of the most common types of arthritis among children. According to JIA guidelines for physical activity (PA), structured PA interventions led to improved health outcomes. However, many PA programs, such as yoga and aerobic dance, have not been studied in this population despite being popular among youth. Web-based PA programs could provide patients with accessible and affordable interventions.

**Objective:**

The primary aims of the proposed pilot randomized controlled trial (RCT) are to examine (1) the feasibility of conducting a full-scale RCT to evaluate the effectiveness of two popular types of PA: a yoga training program and an aerobic dance training program, in female adolescents (aged 13-18 years) with JIA compared with an electronic pamphlet control group; and (2) the acceptability of these interventions.

**Methods:**

A three-arm prospective randomized open-label study with a parallel group design will be used. A total of 25 female adolescents with JIA who have pain will be randomized in a ratio of 2:2:1 to one of the 3 groups: (1) online yoga training program (group A: n=10); (2) online aerobic dance training program (group B: n=10); and (3) electronic pamphlet control group (group C: n=5). Participants in groups A and B will complete 3 individual 1-hour sessions per week using online exercise videos, as well as a 1-hour virtual group session per week using a videoconferencing platform for 12 weeks. Participants from all groups will have access to an electronic educational pamphlet on PA for arthritis developed by the Arthritis Society. All participants will also take part in weekly online consultations with a research coordinator and discussions on Facebook with participants from their own group. Feasibility (ie, recruitment rate, self-reported adherence to the interventions, dropout rates, and percentage of missing data), acceptability, and usability of Facebook and the videoconferencing platform will be assessed at the end of the program. Pain intensity, participation in general PA, morning stiffness, functional status, fatigue, self-efficacy, patient global assessment, disease activity, and adverse events will be assessed using self-administered electronic surveys at baseline and then weekly until the end of the 12-week program.

**Results:**

This pilot RCT has been funded by the Arthritis Health Professions Association. This protocol was approved by the Children’s Hospital of Eastern Ontario Research Ethics Board (#17/08X). As of May 11, 2020, recruitment and data collection have not started.

**Conclusions:**

To our knowledge, this is the first study to evaluate the effectiveness of yoga and aerobic dance as pain management interventions for female adolescents with JIA. The use of online programs to disseminate these 2 PA interventions may facilitate access to alternative methods of pain management. This study can lead to a full-scale RCT.

**International Registered Report Identifier (IRRID):**

PRR1-10.2196/12823

## Introduction

### Juvenile Idiopathic Arthritis and Physical Activity

Juvenile idiopathic arthritis (JIA) is one of the most common types of arthritis among children [1-3]. Symptoms, which include joint pain, stiffness, swelling, and fatigue [4-6], can significantly affect most children’s health-related quality of life (HRQOL). Although adolescents in the general population spend on average 57% of their time engaging in sedentary behavior after school [7], youths with JIA tend to be less active than their healthy peers and meet the national recommendations for physical activity (PA) less often [8]. Physical inactivity associated with JIA may lead to poor bone mineral density, increased fatigue, decreased muscle strength, reduced cardiorespiratory fitness capacity, reduced endurance, loss of functional independence, and diminished quality of life [9-13]. Prolonged physical inactivity may increase the risk of coronary heart disease, diabetes, hypertension, and obesity in adolescents with JIA [14,15]. It is important to help ensure that these symptoms are managed and to promote PA to reduce these long-term consequences [16]. According to recent guidelines for PA in JIA based on a systematic review of the literature [16], structured PA interventions compared with a control were, in general, effective in the self-management of JIA for numerous health outcomes, such as a reduced number of active joints [17,18], diminished pain intensity [19], increased joint range of motion [18,19], increased muscle strength [20,21], improved functional status [19,22], and enhanced quality of life [19,22].

### Yoga and Aerobic Dance

Many specific PA programs, such as yoga and aerobic dance, have not yet been studied in this population [16] despite being recommended by several rheumatology organizations such as the Arthritis Foundation and being popular among youths, especially females [23,24]. The fact that yoga and aerobic dance appeal to female adolescents may encourage them to adopt and adhere to these programs longer [23-25].

Yoga has been found to be beneficial for adults of both sexes with rheumatoid arthritis (RA) in reducing pain, pain disability, and swollen and tender joints, increasing functional status and enhancing quality of life, general health, and vitality as well as self-efficacy regarding pain [26-29]. Although no randomized controlled trial (RCT) has demonstrated the effectiveness of yoga in the management of JIA, a single-case design study found yoga (practiced by following a DVD) to be safe and feasible for 2 female adolescents with JIA [25].

There are currently no RCTs on the effectiveness of structured aerobic dance programs for the management of JIA, although it was found that a Zumba program improved the quality of life (*P*<.001) and aerobic cardiovascular capacity (*P*<.001) of female college students aged between 19 and 23 years from the general population [30]. Other studies showed that a dance intervention improved self-rated health and reduced emotional distress among adolescent girls with internalizing problems (eg, depressed mood, low self-worth) [24,31].

### Disseminating Physical Activity Through Information and Communication Technologies

Although PA interventions may be effective, there is a need to disseminate them to patients in a feasible and cost-effective way. A recent systematic review [32] revealed that information and communication technologies (ICTs) such as a Facebook offer for an exciting dissemination strategy by providing users continuous self-monitoring and self-management as well as real time feedback and information exchange [33-36]. ICTs are particularly appealing to young people as they enhance accessibility and acceptability [34,37-39]. Although ICTs have been successfully used to engage the youth, change behaviors, and provide support and education [32,39-42], few RCTs have used them to deliver information on health-related interventions [43-47]. One study successfully delivered videos of effective PA interventions to adults with arthritis via Facebook [48]. Facebook is one of the most popular and frequently used social media platforms among adolescents [34,38,39,41,49,50]. It has many features that can collect data on content popularity and audience response [51,52]. However, a similar RCT has not been conducted in JIA. Given the high prevalence of JIA among females (ie*,* 2-4 times more than males) [2,53,54], there is a need to disseminate potentially effective PA activities such as yoga and aerobic dance to this population in an accessible and affordable manner.

### Objectives

The primary objectives of the proposed single-blind three-arm pilot RCT with a parallel group will be to examine (1) the feasibility of a 12-week full-effectiveness trial of two popular types of PA and an electronic pamphlet control group: group A: an online yoga training program and a PA electronic educational pamphlet; group B: an online aerobic dance training program and a PA electronic educational pamphlet; group C: an electronic pamphlet control group (PA electronic educational pamphlet) postintervention; and (2) the acceptability of the yoga and aerobic dance PA interventions. The secondary objective of this pilot RCT will be to examine the initial effectiveness of a yoga training program (group A) and an aerobic dance training program (group B) on pain intensity, participation in PA, morning stiffness, functional status, self-efficacy, fatigue, patient global assessment, disease activity, and adverse events in female adolescents (aged 13 to 18 years) with JIA compared with an electronic pamphlet control group (group C) postintervention.

## Methods

The following methodology is in full agreement with the Standard Protocol Items for Randomized Trials recommendations for RCTs [55-57] to ensure methodological rigor.

### Study Design

This study will use a three-arm prospective randomized open-label study. Participants will be randomized to one of the 3 following groups in a ratio of 2:2:1: group A: an online yoga training program (and a PA electronic educational pamphlet; n=10); group B: an online aerobic dance training program (and a PA electronic educational pamphlet; n=10); and group C: an electronic pamphlet control group (PA electronic educational pamphlet; n=5). The total intervention period will be of 12 consecutive weeks. The frequency and duration of the interventions are based on other trials of yoga and PA interventions in pediatric and adult rheumatology [18,20,22,26,58]. As this pilot RCT involves physical interventions (ie*,* yoga and aerobic dance training programs), the participants and the research coordinator administering the program will not be blinded. However, the use of anonymous online self-reported questionnaires administered at baseline and postintervention will reduce potential detection bias. With training and standard operating procedures in place, it is anticipated that any performance bias due to not blinding will be minimized.

### Study Population

Participants will be included in the study based on the following criteria: (1) female adolescents aged between 13 and 18 years; (2) diagnosis of JIA by a rheumatologist according to the International League of Associations for Rheumatology (ILAR) criteria; (3) absence of serious comorbidities or chronic diseases or of chronic pain that is unrelated to JIA (eg*,* cancer, fibromyalgia), which may impact the ability to understand and use the exercise program or complete outcome assessments (as determined by the treating rheumatologist); (4) presence of arthritis-related pain during regular activities of at least 30 on a 100-mm visual analog scale (VAS) in the past month; (5) JIA-specific medication regimen not expected to change during the study period; (6) self-reported as not meeting Health Canada’s and American College of Sports Medicine’s guidelines for PA (<60 minutes of moderate-to-vigorous PA per day) and not using physical interventions/treatments other than medication prescribed for JIA pain relief or over-the-counter medication; (7) capable of using and accessing the internet weekly for the study duration; (8) no contraindications to exercise (according to the treating rheumatologist); and (9) ability to understand English. Participants will be excluded from the study if they do not meet the inclusion criteria.

### Interventions

#### Group A

Participants assigned to this group will be invited to complete a yoga training program. The yoga training program, an evidence-based educational program, is a structured low-intensity Vishwas Raj [26]. This yoga program consists of stretching, strengthening, meditation, and deep breathing and has been shown to be acceptable and effective among adults with RA. Participants will be asked to complete 3 individual 1-hour sessions per week for 12 consecutive weeks by watching a previously filmed session led by a qualified yoga instructor and posted on Facebook. They will also take part in a 1-hour virtual group session per week using a video-conferencing platform. Participants will also participate in weekly online consultations with a research coordinator and discussions on Facebook with other participants in group A. Finally, participants will receive an electronic pamphlet on physical exercise developed by the Arthritis Society. The electronic pamphlet, which groups B and C will also receive, explains how PA can help manage arthritis. It includes valuable tips and a detailed list of the various types of PA and exercises.

#### Group B

Participants assigned to this group will receive the electronic pamphlet on physical exercise and will be invited to complete an aerobic dance program. The aerobic dance program is a low-to-moderate intensity–level program and will also use a video. The video, adapted for youths with JIA, was developed with feedback from a JIA patient with experience in aerobic dance and a physiotherapist with experience in pediatric rheumatology. The aerobic dance program will have the same characteristics in terms of frequency, total number of sessions, and total duration as the yoga program (ie*,* three 1-hour individual sessions and one 1-hour virtual group session per week for 12 weeks). Participants will also participate in weekly online consultations with a research coordinator and discussions on Facebook with other participants in group B.

#### Group C

Participants assigned to this group will receive the electronic pamphlet on physical exercise and will participate in weekly online consultations with a research coordinator to discuss their use of all types of PA in the past, the electronic pamphlet on PA, and their future use of PA. They will also participate in discussions on Facebook with other participants in group C. After the completion of the study, the yoga and aerobic dance training videos will be available to all participants, including those in the control group. In recruitment documents, this group will be termed the electronic pamphlet group.

### Outcomes

The feasibility of a full trial will be monitored throughout the study (eg*,* randomization, recruitment and retention of participants, data collection, and self-reported adherence). The acceptability of the interventions will be assessed using online questionnaires at the end of the study.

Clinical outcomes will be evaluated at baseline, weekly, and postintervention (at 12 weeks) through the use of online questionnaires in REDCap (Vanderbilt University). At baseline, pain intensity, functional status, fatigue, and self-efficacy will be assessed. Pain intensity and fatigue will be monitored weekly. Finally, all of these outcomes as well as the patient’s global assessment will be assessed postintervention. Outcomes were selected based on a JIA core set of outcomes to assess in trials [59]. Outcomes that were found to be essential include pain, functional status, patients’ overall well-being (global assessment), joint inflammation signs, and adverse events [59]. Other outcomes that were found to be important but not essential include fatigue, joint stiffness, and PA [59]. Other outcomes need more research to be endorsed, such as coping with the illness, which is related to self-efficacy [59].

### Recruitment

The recruitment of a convenience sample of 25 participants (in a ratio of 2:2:1) will be performed at the Children’s Hospital of Eastern Ontario (CHEO) Pediatric Rheumatology Clinic. Rheumatologists at the CHEO Rheumatology Clinic will be provided with the inclusion and exclusion criteria for the study. During regular scheduled visits, treating rheumatologists will identify and direct eligible participants with JIA (and their parents) to the research coordinator who will explain the study in detail. Eligible participants will be invited to complete a screening questionnaire to ensure that they meet the study selection criteria before central randomization. If eligible, participants will be asked to sign either a consent form if they are able to consent or an assent form and their parent/guardian will be asked to sign a consent form. They will also complete a questionnaire that will gather sociodemographic and medical information as well as a validated questionnaire on participation in PA. They will also be asked about their preference in terms of PA.

### Participant Allocation

Once informed consent is obtained, participants will be randomly assigned to one of the 3 groups using the central randomization scheme [60] and based on a sequence of computer-generated random numbers using a blocking factor (randomly varying between 6 and 9). The central stratified randomization process will be based on age (between 13 and 15 years and between 16 and 18 years).

Before running the randomization program, the data manager will document the participant’s initials (first and last names) and date of birth (month and year). After running the program, the data manager will prepare sealed opaque envelopes with the group allocation and study ID number for each patient and will provide it to the research coordinator. Participants will open the sealed opaque envelope containing their group assignment and a confidential link to access their Facebook group page where further instructions can be found. The research coordinator will explain the procedures related to the group assigned and will ask participants to indicate their availability for an initial individual online consultation as well as for virtual group sessions. Participants will be asked not to change their usual level of PA during the study except for following the program to which they were assigned.

At the clinic, participants will complete a questionnaire that will gather the following information:

- Sociodemographic information: partial date of birth (month/year), age, gender, ability to understand English, and ability to use and access the internet weekly for the study duration.
- Medical information: diagnosis, partial date of diagnosis (month/year), disease duration, presence of serious chronic diseases (eg, cancer or other illnesses) or cognitive impairments, presence of contraindications to exercise (as determined by the treating rheumatologist), current use of JIA-specific medication, anticipated change in JIA-specific medication in the next 12 weeks, and use of physical interventions/treatments other than the prescribed medication for JIA pain relief and pain level when performing regular activities. A chart review will help verify this information.
- PA information: the amount of PA performed weekly using a validated exercise log [61] and preferences in terms of PA (eg, yoga, Zumba, or other types).

At baseline, all the participants will be asked to do the following:

1. Create a new Facebook account: All participants will be asked to create an account specifically for the study and select a username that is meaningful to them but does not contain their first or last name, allowing them to remain anonymous.
2. Access the group Facebook page: All participants will be invited to access the link to their group Facebook page, which they will be provided during their group assignment. Participants will be invited to follow the REDCap link provided on the Facebook page and answer baseline questionnaires about their participation in PA, pain, morning stiffness, functional status, fatigue, self-efficacy, and patient global assessment. This will take about 20 to 30 min to complete. The Facebook page will also contain the electronic pamphlet on PA (for all groups), the exercise videos (for groups A and B), and other information.

Following these steps, participants will be asked to complete the training program:

Groups A and B:

- Participants will be invited via Facebook and email to attend an individual online consultation with the research coordinator using a videoconferencing platform. During this consultation, participants can ask questions related to their group assignment, voice their concerns, and establish a plan of action. For the remainder of the study duration, participants will be encouraged to attend, when possible, individual 10-minute online consultations weekly. During these consultations, participants can provide their feedback and discuss their use of the PA program as well as facilitators and barriers to participating. Private meetings are less difficult to schedule than group meetings and provide greater privacy. They may also be more motivating and less intimidating for participants who may feel their voice really matters and has an impact.
- For the study duration, participants will be asked to complete 3 individual exercise sessions as well as 1 virtual group session each week. The links to the videos will be posted on Facebook and sent by email for participants to use for their individual exercise sessions. For group sessions, the videoconferencing platform link will be posted on Facebook and sent to participants via email. Virtual group sessions will help ensure that they perform the exercises that they observe in the video safely and that they do not hurt themselves. They will also be able to see other participants and be seen if they wish, which will be a motivating factor to perform the PA. A schedule of virtual group sessions offered in the morning or afternoon will be posted on the Facebook group page or sent by email.
- Participants will be asked to record their PA using a validated exercise log [61] as well as their pain intensity and fatigue by completing a brief online questionnaire that regroups valid instruments on a weekly basis. They will be reminded to do so via Facebook, email, and during their online consultations.

Group C: Participants will be invited via Facebook and email to attend an individual online consultation with the research coordinator using a video-conferencing platform. During this consultation, participants can ask questions related to their group assignment, voice their concerns, and discuss PA in general and their plan for PA in the future. They will also be invited to consult the electronic pamphlet on PA each week. For the remainder of the study duration, participants will be encouraged to attend, when possible, individual weekly 10-min online consultations. During these consultations, participants can discuss their use of PA, as well as facilitators and barriers to participating in PA. Participants will be asked to record their PA using a validated exercise log [61] as well as their pain intensity and fatigue by completing a brief electronic questionnaire that regroups valid instruments on a weekly basis.

Throughout the study, the Facebook pages will provide a means of communication for participants who will be able to share their experiences, post comments on the videos, and ask questions of each other and the research coordinator. Access to Facebook group pages will be restricted to study participants. Adolescents who cannot or do not want (or are not granted parental permission) to access Facebook will be given a link to the CHEO webpage that will contain information related to the study and their group. However, they will not be able to interact (ie*,* leave comments or questions) using this platform.

At 6 and 12 weeks, all participants will be asked to complete online questionnaires about the program that they were allocated to as well as the acceptability of Facebook to deliver PA programs. They will also be asked if they used any other PA during the study. They will also complete online questionnaires assessing their pain intensity, functional status, fatigue, self-efficacy, and well-being (using a patient global assessment). This will take about 20 to 30 minutes to complete.

As a compensation for the time given by participants, they will receive a US $21.40 gift certificate for completing baseline questionnaires and a US $21.40 gift certificate for completing postintervention questionnaires. They will also receive a personalized certificate of participation (community hours for school).

### Outcome Measures

The electronic questionnaires will be made accessible to all participants on each group's Facebook page with a URL link or on the CHEO webpage for those who do not wish to create a Facebook account. Participants in groups A, B, and C will be emailed the same URL link to access the questionnaires. Using the *wall* on the Facebook page for all groups, or the CHEO webpage, the research team will provide updates and reminders to all participants regarding deadlines to complete the questionnaires.

The following outcome measures will be used to answer the primary objective:

1. Feasibility will be assessed by the recruitment rate, the randomization process, and self-reported adherence and measured using a questionnaire, as well as protocol deviations, dropout rates, and percentage of missing data in the questionnaires.
  Recruitment rate: The number of participants recruited in the trial and the time used for recruitment will be documented in a feasibility form. The number of participants will be divided by the duration of recruitment. This will be measured on a continuous basis for up to 12 weeks.
  Randomization process: The ease at which the randomization process is conducted will be described by researchers in a feasibility form. This will be measured on a continuous basis for up to 12 weeks.
  Number of protocol deviations: The number of protocol deviations, the time at which they occur, the group in which they occur, and the potential reasons for these will be documented in a feasibility form. Participants will be asked why they deviated from the protocol by researchers using a feasibility form. This will be measured on a continuous basis for up to 12 weeks.
  Dropout rates: The number of participants who dropped out of the trial, the time at which they dropped out, the group in which they belonged, and the reasons for these will be documented in a feasibility form. Participants will be asked why they dropped out by researchers. The number of dropouts in each group will be divided by the duration of the trial. This will be measured on a continuous basis for up to 12 weeks.
  Percentage of missing data in the questionnaires: The percentage of missing data in the questionnaires, the time at which they occur, and potential reasons for these will be documented in a feasibility form. This will be measured on a continuous basis for up to 12 weeks.
  Self-reported adherence: Self-reported adherence to the training programs will be assessed by monitoring the number and length of sessions and dividing the time spent performing the program by the duration of prescribed sessions of either yoga or aerobic dance. Self-reported adherence will be recorded weekly using an adapted online version of the validated 7-day PA Report calendar (in minutes) [62]. This will be measured each week for up to 12 weeks.
2. The acceptability of the yoga or aerobic dance interventions will be assessed using a questionnaire-administered postintervention. Questions will explore the acceptability of online consultations, virtual group sessions, PA videos (either yoga or aerobic dance programs), electronic pamphlet, and training programs (content and duration of the yoga and aerobic dance programs), as well as facilitators and barriers to participating. This questionnaire will be administered at 6 and 12 weeks.
3. The usability of Facebook will be assessed by the System Usability Scale (SUS) [63,64]. The SUS is composed of 10 items using a 5-point scoring system ranging from Strongly Disagree (a score of 1 out of 5 for each question) to Strongly Agree (a score of 5 out of 5 for each question). The total score ranges from 5 (low usability) to 50 (high usability). The usability will be measured at 6 and 12 weeks.
4. The usability of the videoconferencing platform will be assessed by the SUS [63,64]. The SUS is composed of 10 items using a 5-point scoring system ranging from Strongly Disagree (a score of 1 out of 5 for each question) to Strongly Agree (a score of 5 out of 5 for each question). The total score ranges from 5 (low usability) to 50 (high usability). The usability will be measured at 6 and 12 weeks.
5. The use of Facebook will be assessed by the number of posts, live online discussions, and views of the videos. The use will be measured at 6 and 12 weeks.

The following outcome measures will be used to answer the secondary objective:

1. *Pain intensity* will be assessed using the electronic Childhood Health Assessment Questionnaire (CHAQ) 100-mm pain VAS subscale [65]. It uses a scoring system where 0 mm represents no pain and 100 mm represents very severe pain the past week. This measure has already been validated in the JIA population [65,66]. Pain intensity will be measured at baseline, 6 weeks, and 12 weeks.
2. *Participation in PA (level of PA)* will be assessed by using the electronic Physical Activity Questionnaire for adolescents (PAQ-A) aged 14 to 18 years, a 7-day recall instrument measuring the level of PA within the last 7 days [67-69]. The PAQ-A is composed of 8 items using a 5-point scoring system. The total score is calculated by taking the mean score out of 5, with 1 indicating low PA and 5 indicating high PA. The PAQ-A has been reported to be a valid and reliable measure of general PA levels in youths and adolescents [67-69]. Participation in PA will be measured at baseline, 6 weeks, and 12 weeks.
3. *Participation in PA (duration and intensity)* will be assessed by using an exercise log, based on the 7-day PA Recall calendar [62], which will be used weekly. The PA level will be reported in terms of minutes and intensity levels (3 subscales: moderate, hard, and very hard intensity; subscale total scores in minutes) and will be transformed into the metabolic equivalent of tasks, which are units of the basal metabolic rate and express the energy cost of PA. Participation in PA will be measured at baseline, 6 weeks, and 12 weeks.
4. *Morning stiffness* will be assessed by using electronic self-reported questions asking about the presence (Yes/No) and duration of morning stiffness in minutes. It will be measured at baseline, 6 weeks, and 12 weeks.
5. *Functional status* will be assessed using the electronic CHAQ. The CHAQ contains 8 domains (dressing/grooming, arising, eating, walking, hygiene, reach, grip, and activities), and items are scored using a 4-point Likert scale of 0 to 3, where 0 represents the ability to perform the activity with no difficulty, 1 represents the ability to perform with some difficulty, 2 represents the ability to perform with much difficulty, and 3 represents the inability to perform over the past week. The mean of the 8 scores will determine the total CHAQ score ranging from 0 to 3, with a lower score indicating higher functional status and a higher score indicating lower functional status. It is the most widely used functional health status measure in JIA, which is a reliable and valid tool for the functional, physical, and psychosocial assessment of children with JIA [65]. It will be measured at baseline, 6 weeks, and 12 weeks.
6. *Fatigue* will be assessed by using an electronic version of a subset of the Patient-Reported Outcomes Measurement Information System Pediatric Short Form version 1.0—Fatigue 10a [70], which measures fatigue and its impact, scored from 1 to 5, where 1 represents never tired and 5 represents almost always tired in the past week. It will be measured at baseline, 6 weeks, and 12 weeks.
7. *Self-efficacy* will be assessed by using the Children’s Arthritis Self-Efficacy Scale (CASE), a specific, valid and reliable tool for JIA [71]. CASE is an 11-item self-report scale that is divided into 3 concepts: activity, symptom, and emotion. A 5-point Likert scale is used to rate responses for each item, where 1 represents not at all sure and 5 represents very sure based on the confidence of the child to manage disease effects. It will be measured at baseline, 6 weeks, and 12 weeks.
8. *Patient Global Assessment* will be assessed by using an item used in the Juvenile Arthritis Quality of Life Questionnaire (JAQQ). JAQQ was designed to assess HRQOL in children aged 2 to 18 years JIA or juvenile spondylarthritides [72-75]. JAQQ includes 4 subscales (gross motor function, fine motor function, psychosocial function, and systemic symptoms) as well as a pain and patient global assessment. The patient global assessment asks how youths have been since the last assessment (prior week) on a 5-point Likert scale, scored from 1 to 5, where 1 represents much better and 5 represents much worse. It will be measured at 6 weeks and 12 weeks.
9. *Disease activity* will be assessed by using the active joint count, the number of joints with active disease according to the rheumatologist [1].
10. Adverse events will be reported by participants in answers to open questions.

### Data Analysis

Descriptive statistics, such as proportions, means, and standard deviations, will be used to summarize the baseline and end-of-study variables across the 3 study groups (groups A, B, and C) and to assess the distributional assumptions of the statistical techniques used. For the primary objective, data on recruitment rate, randomization process, interventions, clinical and ICT outcome measures, dropout rates, and adherence rates for the interventions will be collected. For the secondary objective, a repeated measures analysis of variance with the fixed-factor study group (A, B, or C) and the within-factor assessment time (0, 6, and 12 weeks) will be conducted to compare groups for the clinical outcome measures at 0, 6, and 12 weeks. An intention-to-treat analysis will be conducted and mixed model repeated measures will be considered to accommodate missing data.

### Sample Size Calculation for Future Full-Scale RCT

The current sample size is adequate for pilot studies involving group comparisons to assess feasibility and to estimate the variance to determine the sample size for a definitive trial [76].

## Results

This pilot RCT has been funded by the Arthritis Health Professions Association (AHPA). This protocol was approved by the CHEO Research Ethics Board (#17/08X). As of May 11, 2020, recruitment and data collection have not yet started.

## Discussion

### Strengths

This multidisciplinary and multidimensional rehabilitation study is a rigorous, accessible, single-blind, three-arm pilot RCT using the prospective randomized open blinded endpoint design. It involves web-based, innovative, accessible self-management programs for adolescents with JIA similar to those used in previous studies [41,77,78]. The first strength of this study design is that it will include PA activities that are recommended by several rheumatology organizations and may meet adolescent females’ activity preferences. To our knowledge, this is the first study to assess the effectiveness of aerobic dance and yoga on pain management in female adolescents with JIA. It also aims to reduce daily pain intensity and fatigue severity and improve the functional status, self-efficacy, and PA adherence among female adolescents with JIA. The second major strength of this study is that it will use ICTs to deliver these activities and information to youths. The rapid increase in eHealth will appeal to young participants who are already consulting online resources for self-management [79]. This design will be able to overcome time, geographical, and financial constraints. Furthermore, online questionnaires will increase the accessibility and confidentiality of collected outcome measures.

The involvement of a research coordinator, who will be mentored by a team of rehabilitation specialists, is a strength of the study. Rehabilitation specialists play an essential role in supporting community-based self-management programs targeting youths living with chronic conditions, such as JIA [16].

Separate Facebook group pages and individual online consultations using a video-conferencing platform minimized the potential risk of contamination between comparison study groups. Central randomization is ideal for minimizing the potential risk of biases. This proposed pilot RCT is necessary to address the questions of clinical and scientific importance for rehabilitation specialists in improving the health of female adolescents with JIA through the use of PA-based interventions.

### Challenges (or Weaknesses)

The blinding of participants is impossible in this type of study, as is generally the case with physical rehabilitation RCTs [80].

There is an increased risk of Type 1 error, rejecting the null hypothesis when it is actually true, due to the presence of multiple outcomes (ie, multicollinearity). The results of this study will likely only be generalizable to female adolescents (ie*,* those aged 13 to 18 years) with JIA who consult at a pediatric rheumatology clinic in a tertiary care center.

Due to financial limitations, we will use low-cost, accessible ICTs. Although a virtual gym with a live PA instructor would be ideal, participants will have access to recorded sessions led by PA instructors and approved by rehabilitation specialists in rheumatology and will receive support from a research coordinator trained in PA at least once a week.

Hospital-based recruitment may attract more severe cases of JIA. However, central randomization will be implemented as it is ideal to minimize the potential risk of biases.

This study will examine the effectiveness of yoga among female adolescents with JIA, as outcomes will be assessed at 12 weeks.

#### Population

To minimize the potential misclassification bias, rheumatologists involved in this project will ensure that each participant enrolled in this pilot RCT has a diagnosis of JIA, using ILAR criteria [81].

Although participants cannot be blinded to the group assignment given the nature of the intervention, the investigator who will analyze the data will be blinded.

#### Intervention

Aerobic dance [30] and yoga exercise programs [26-28,58] are promising for managing JIA pain in young women because they are popular among female adolescents [23-25,30]. These programs will be accessible and inexpensive through Facebook and YouTube [51,79,82].

#### Comparator

The use of an electronic pamphlet control group is acceptable as they will have access to Facebook and will have online consultations on PA. Furthermore, participants will receive the same yoga or aerobic dance exercise videos that were offered to the intervention groups after study completion.

#### Outcomes

Assessments will include a range of clinical outcome measures validated in JIA. Although pain and other outcomes are self-reported, they represent important unwanted symptoms that have functional consequences that affect the activities of daily living of youths with JIA. Furthermore, objective measures of these symptoms are difficult to obtain. However, it is important to be mindful that self-reported measures may lead to reporting bias.

There is also the potential to include further measurements such as PA level (using a questionnaire or an accelerometer) and aerobic exercise testing (ie*,* assessment of submaximal oxygen consumption: PWC_170_, physical working capacity at a heart rate of 170 beats per min) to determine potential differences between treatment arms in the full-scale RCT.

#### Time

The study duration will be 12 consecutive weeks. The frequency and duration of the intervention is based on other trials of yoga and PA interventions in pediatric and adult rheumatology. This length is sufficient to see improvements among adolescents.

Providing participants with weekly online consultations with the research coordinator and providing them with 1 virtual group session each week will help ensure that the participants perform the exercises properly and may also potentially facilitate adherence.

If the proposed PA interventions are effective, we will produce videos to enrich the People Getting a Grip program, an online educational program comprising informational videos and practical videos that provide information on various PA programs for arthritis. These videos will also be freely available on the Arthritis Society webpage and could eventually be used for the full-scale RCT.

### Conclusions

This pilot RCT has substantial potential to enhance our understanding of the potential effectiveness of popular PA interventions disseminated to patients with JIA using ICTs, making it a novel study. This will help understand which PA intervention has the most potential for improved outcomes. It will also help determine whether these PAs and ICTs are acceptable to youths with JIA and whether a larger trial would be feasible. The proposed pilot RCT will contribute to the knowledge of the effect of a specific functional activity on self-reported pain relief, functional status, and self-efficacy as well as PA adherence, daily pain, and fatigue intensity.

This study could also set the standard for community-based care for female adolescents with JIA. Moreover, other rehabilitation or functional interventions could be examined for youths with other chronic diseases**.**

## Appendix

Multimedia Appendix 1 Peer Review Report AHPA.

## Abbreviations

AHPA: Arthritis Health Professions Association
CASE: Children’s Arthritis Self-Efficacy Scale
CHAQ: Childhood Health Assessment Questionnaire
CHEO: Children’s Hospital of Eastern Ontario
HRQOL: health-related quality of life
ICT: information and communication technology
ILAR: International League of Associations for Rheumatology
JAQQ: Juvenile Arthritis Quality of Life Questionnaire
JIA: juvenile idiopathic arthritis
PA: physical activity
PAQ-A: Physical Activity Questionnaire for adolescents
RA: rheumatoid arthritis
RCT: randomized controlled trial
SUS: system usability scale
VAS: visual analog scale
