# Supplementary material for: Yoga and Aerobic Dance for Pain Management in Juvenile Idiopathic Arthritis: Protocol for a Pilot Randomized Controlled Trial
Source: JMIR Res Protoc. 2020 Jul 6;9(7):e12823. doi: 10.2196/12823 (PMC7381073; doi:10.2196/12823)
Supplement: Multimedia Appendix 1 [file resprot_v9i7e12823_app1.pdf]

AHPA Grant Proposal Review 2015

Title:
"Are popular structured physical activity programs promising for the pain management of Juvenile Idiopathic Arthritis? A pilot randomized controlled trial"

Principal Investigator:
Lucie Brosseau

Co-investigators:
Karine Toupin-April, Sabrina Cavallo, Jennifer Stinson, George A. Wells

---

Aggregate Comments From All Reviewers

Strengths and Weaknesses of the Proposal

Does the review of literature support the study purpose, design and methods?

Reviewer 1: This is a nicely written proposal that looks at the feasibility of implementing exercise program (Yoga, Zumba, waiting list control groups) for adolescent girls with Juvenile Idiopathic Arthritis (JIA). Current state of the knowledge was presented with appropriate references. The authors provide a strong justification of proceeding with a feasibility study on this topic.

Reviewer 2: There is extensive literature supporting the applicant's premise, including publications in this area by the applicants and other researchers.

Reviewer 3: The authors have conducted a systematic review and determined there is no evidence for Yoga and Zumba in this population. They complete focus groups and established interest.

Reviewer 4: This pilot proposes to answer a number of questions that will lead to the development of a successful full scale RCT to adequately answer the main question.

Reviewer 5: Very straightforward and clear objectives with achievable outcomes.

Reviewer 6: Nice review of the literature that provides a succinct rationale as to why yoga and Zumba are relevant physical activities to test with this population, and also why there may be merit in using Facebook and Skype as dissemination channels.

Reviewer 7: Rationale is sound with adequate lit support.

Are the methods of data collection appropriate to the design of the study?

Reviewer 1: This is a well planned study with attainable objectives. Within the context of a feasibility study, the authors are looking at many relevant items that inform a formalized RCT. The authors may wish to define the different types of JIA within this sample as some participants may respond differently than others. The primary outcome is pain; however, that authors may wish to measure fatigue as a major factor in participating in the exercise programs. It is unclear what are the back-up plans if few

participants volunteer via the advertisements on TAS Facebook. The authors are to be commended on selecting an activity measure that derive METs. All too often physical activity measures are selected that categorize intensity of activity without using METs.

Reviewer 2: Methods are clear, though no physical or virtual face time with participants before the study begins may be a drawback. Concern about FP involvement in signing off on serious co-morbidities re: timing.

Reviewer 3: The research objectives were to 1) to examine the feasibility of a non-aerobic physical activity (PA) intervention (i.e. a Yoga raining program) and an aerobic PA intervention (i.e. Zumba training program) on pain relief in female adolescents (14 to 18 years old) with juvenile idiopathic arthritis (JIA) compared to a waiting list control and 2)to examine the initial effectiveness of these interventions. The study is well designed. My only concern is that it is not clear whether this is a feasibility study or a pilot study. There are different purposes for these. Furthermore, effect of intervention should not be an objective. There is insufficient power.

Reviewer 4: The use of social media is relatively new in engaging people in health promoting activities. Youth use this form of communication with ease and comfort. It is noted that participants will rate themselves as inactive and I therefore have concerns about any program that is self-directed with outcomes that are self reported in a group who may need motiviation. I do not see any anything in place to ensure that participants are indeed participating. Involvement of a second person to ensure adherence may ensure that full participation is undertaken. As well, participation in the one on one wwekly on line meetings is not mandatory but is only available.

Reviewer 5: Overall good, but missing some key information such as justification for 6 week intervention. I also do not agree that it is double-blind. Importantly, the applicants have not convinced me that they can prevent cross-contamination of groups given that info will be online freely available (eg. youtube videos).

Reviewer 6: Nice tight study. Small quibbles: seems odd to have an OT as the expert and not a PT familiar with both yoga and Zumba; will the researchers have access to all parts of the Facebook posts, and if so, this should be stated; the Facebook posts and Skype transcrips would provide valuable information about acceptability and barriers to use (of both the physical activity and the ICTs), yet there does not seem to be a plan to analyze this data; and I'd sell the Skype sessions to participants as a 'touch base' where they can share what they like and don't like about their assigned PA intervention. The points raised in the study can be probes, but currently the Skype sessions look more like "checking up" on participants rather than "checking in" with participants.

Reviewer 7: Recruitment bias is inevitable to participants who have access to the IT needed, and who are more active in the cyber media. But this weakness may also be a strength as the study participants may be more likely less physically active when compared to those who seldom show up in the cyber media.

Will the research generate new knowledge?

Reviewer 1: An intriguing component of this study is use of social media for delivery and meetings. This is a strength of this study that will generate new and practical information to the area of physical activity in patient populations. It will be challenging to monitor whether there is cross-contamination

with other participants in other groups of the study. Because this is a feasibility study, the authors may wish to explore how the social media may be monitored between groups.

Reviewer 2: Researchers state there is little research into physical activity in female teen JIA. New ground.

Reviewer 3: Long term goal is to design a large RCT. The results of RCT will generate new knowledge. This is the first step.

Reviewer 4: If full participation occurs, logs kept and questionnaires completed considerable knowledge could be generated not only in regards to the relevance of yoga and zumba as part of an exercise regime but in communication strategies in working with adolescents.

Reviewer 5: Unclear why Yoga and Zumba is "recommended by several arthritis organizations for JIA" if there is no evidence. Is this novel or not?

Reviewer 6: This pilot study will provide useful data that can be used to support funding for a full RCT. With the addition of a small qualitative component, valuable data can also be gleaned on how to improve the intervention on its next iteration.

Reviewer 7: Feasibility is an important question to ask when one considers the use of social media to deliver health information. This study asks questions relating to recruitment rate, and more importantly, adherence and drop-out rate, which will generate some fundamental data for reference.

Is the proposed research relevant to people with arthritis?

Reviewer 1: Investigation of physical activity is warranted within arthritis population. Interventions to improve physical activity is much needed. Although this study looks at a rather small segment of those people with arthritis, adolescent girls 14-18 years of age, the intervention could be an effective approach to encouraging physical activity not only in this group but also with other types of arthritis.

Reviewer 2: A very relevant and a socially attractive way to engage teens with JIA in developing a habit of increased physical activity.

Reviewer 3: No comments.

Reviewer 4: Patients with arthritis are best served when they learn in the early stages how to take charge of their own health and see themselves as participants in their own healthcare.

Reviewer 5: Directly relevant.

Reviewer 6: Low levels of physical activity and increasing childhood obesity rates makes it imperative to pursue these types of studies.

Reviewer 7: No comments.

Is the study feasible within timeline and budget?

Reviewer 1: For a feasibility study the budget looks reasonable. Given the proposed study, this is a team that is very well qualified to successfully complete the study and build upon the findings.

Reviewer 2: It's a small sample and a low budget, yet researcher's enthusiasm may push it to completion, especially with good and speedy recruitment.

Reviewer 3: The budget is reasonable and feasible.

Reviewer 4: Well considered.

Reviewer 5: Pilot study that should be finished with no problem.

Reviewer 6: Timeline appears sufficient. It's hard to conceive that this study can be done with $5K but one couldn't pare this study down much more than it currently is.

Reviewer 7: No comments

Final Summary

Reviewer 1: The strength of the application is the relevance and intervention looking a physical activity in JIA. The social media component opens many different applications to the PA intervention. Lastly, a strength of this application is the team itself with both experienced and new investigators who have complementary backgrounds. The authors may wish to think about the primary outcome in more detail given that fatigue and aerobic capacity may be factors that improve with adherence to the intervention.

Reviewer 2: On the plus side the interventions chosen will interest girls in the 14 to 18 age group, and the researchers conducted a focus group to verify this. The girls on the waiting list are not disadvantaged because they will ultimately have access to the same interventions. (Question: Would you leave the facebook groups available to the teens who participated and for the control group?) I like that the patients will meet with a real person from the study weekly online. That way they can ask the OT questions and be encouraged by the other girls. There are good incentives for the girls to stay engaged since they get a gift card for each survey (Paid after they complete both of them might be wise) and community hours as a benefit, plus they have an opportunity to learn to manage their JIA better. If the CHAQ is the juvenile version of the HAQ score questions for adults it seems to be a very blunt instrument to measure change (in my opinion). Maybe the parents should not be included in the whole skype meeting each week, or the OT should suggest at some point the adults to leave in the interest of peer group sharing. On the negative side it may be time consuming to get the OKs from the Family Doctors, and the consents from the parents for the recruits. Also I doubt a lot of teen girls will be frequenting the FaceBook page of the Arthritis Society, which is mentioned as a recruitment strategy. You might need to focus on other more possible methods to recruit them. The dissemination plan is weak in not including the girls as part of the plan. They will circulate information about this faster than the researchers can, and more widely, and may be a source of recruits for the RCT. It's the girls with JIA who are supposed to be the benificiaries of this research. If you have a good bunch in the pilot maybe they would have suggestions for refining your measurement tools or they could suggest a few more questions that may give more insight. Then they will feel more engaged.

Reviewer 3: No summary provided.

Reviewer 4: I believe the reliability of the data may be in question. If the study was reworked to have a system of checks and balances it may be workable. If there is a system in place that I have missed please highlight it or address how you would ensure that the reporting and the participation is accurate.

Reviewer 5: This is a very good proposal that is consistent with the scope of these funds. Future directions are strong and achievable. Some details regarding methods are missing, and I have concerns about the ability to prevent cross-contamination of groups (given that videos will be posted online) and the potential beneficial effect of the Facebook group as a "support mechanism" that can influence results (indeed, it is run by an OT, so there is potential for additional "education treatment"), but the study is otherwise sound.

Reviewer 6: Nice overall pilot study that will provide useful data for a subsequent RCT application. I'd strongly suggest the addition of a qualitative data component so that even if the primary or secondary study outcomes are less positive than expected, or should problems arise in carrying out this study, the researchers will have valuable information for which to re-tool the next iteration of this intervention.

Reviewer 7:

The study results have direct impact to patients with arthritis. The long term disease burden is huge to our society, when considering IA on its own, together with the potential development of other chronic diseases in this group of young people. As younger generation immerse most of their time in the cyber media, using similar pathway to reach out to this group may also be more effective; the intervention in the longer term, may also be very cost-effective.

PA, if conducted appropriately and consistently will likely control and delay many potential chronic diseases that the group with JIA is at risk of developing. This study has strong rationale with good literature support. May need to have additional access code to avoid potential cross contamination where participants of control group access information of the experimental group. Or that group A try doing exercise of group B. Potential particpants who have limited access to computer, internet may also be left out with the current recruitment process.
